# The scope of physiotherapy in men’s health: a qualitative study on clinician experiences

**DOI:** 10.1016/j.clinsp.2026.101039

**Published:** 2026-07-01

**Authors:** Umit Erkut, Birol Önal, Sefa Unes

**Affiliations:** a Rumeli University, Department of Physiotherapy and Rehabilitation, İstanbul, Türkiye; b Atatürk University, Department of Physiotherapy and Rehabilitation, Erzurum, Turkey; c Bingöl University, Faculty of Physical Therapy and Rehabilitation, Bingöl, Türkiye

**Keywords:** Men’s health physiotherapy, Pelvic health, Physiotherapist, Patient perspectives, Clinical practice

## Abstract

**Introduction:**

Men’s health physiotherapy is an expanding subspecialty that addresses pelvic floor dysfunction, sexual health disorders, and chronic pelvic pain in men. However, practitioners’ perspectives remain underexplored, particularly in healthcare contexts where referral pathways and interdisciplinary collaboration are not yet well established.

**Aim:**

This study aimed to explore how physiotherapists experience clinical practice in men’s health physiotherapy and how sociocultural norms and healthcare system structures shape their professional practice.

**Methods:**

In this qualitative study, semi-structured interviews were conducted with ten male physiotherapists working in men’s health physiotherapy in Türkiye between February and March 2025. Interviews were transcribed verbatim and analyzed using Braun and Clarke’s reflexive thematic analysis approach.

**Results:**

Participants reported entering this field through a combination of responding to unmet clinical needs and seeking professional differentiation within a still under-institutionalized area of practice. Clinical encounters were strongly influenced by masculinity-related stigma and privacy concerns, positioning communication and trust-building as central components of care. Although participants valued a holistic approach integrating pelvic health and musculoskeletal perspectives, limited multidisciplinary collaboration and constrained referral pathways restricted the implementation of coordinated biopsychosocial care. Consequently, holistic care was often sustained through individual clinician effort rather than supported by institutionalized interdisciplinary frameworks.

**Conclusion:**

Men’s health physiotherapy practice in Türkiye is shaped by the interaction of professional expectations, masculinity norms, and the structural configuration of healthcare delivery. Formalizing referral mechanisms, strengthening interdisciplinary collaboration, and developing structured educational pathways may support more sustainable and integrated service provision.

## Highlights

Men’s health physiotherapy is a developing professional identity field.
Physiotherapists negotiate masculinity stigma to build trust.
Isolated holism shapes holistic care in clinical practice.
Sociocultural and structural factors shape clinical practice.
Professional roles reflect opportunity-constraint dynamics.

## Introduction

Men’s health physiotherapy is a specialized area of physiotherapy that focuses on the assessment and treatment of conditions related to the pelvic floor, bladder, bowel, and sexual health in men^1^ Physiotherapists specializing in men’s health provide care across diverse clinical settings, managing conditions such as pelvic floor dysfunction, post-surgical recovery, musculoskeletal issues, and chronic pelvic pain. Treatment methods typically include pelvic floor muscle training, manual therapy, neuromuscular re-education, biofeedback, and patient education. These interventions not only help relieve symptoms and prevent complications but also support overall recovery^2^ Beyond physical benefits, men’s health physiotherapy plays a critical role in enhancing psychosocial well-being by improving confidence, reducing anxiety, and helping patients regain a sense of normalcy. When integrated into multidisciplinary care, it offers individualized and comprehensive support tailored to each patient’s needs^3^

Despite the high prevalence of sexual dysfunctions in men, ranging from 10% to 52%, men’s pelvic health remains significantly underrecognized and underdiscussed^4^, ^5^, ^6^ Societal norms and longstanding taboos surrounding male sexual and pelvic health often result in silence, stigma, and reluctance to seek care. Furthermore, the dominance of women’s health in pelvic physiotherapy education and practice has contributed to the underrepresentation of male-specific conditions^7^ Consequently, many men remain unaware of physiotherapy-based treatment options, leading to delayed diagnosis, persistent symptoms, and diminished quality of life^5^

In recent years, the role of physiotherapy in managing pelvic floor dysfunction, particularly incontinence and erectile dysfunction, has received growing attention^8^^,^^9^ This increasing recognition has broadened the scope of men’s health physiotherapy to include musculoskeletal disorders and chronic pelvic pain, reflecting its multidisciplinary nature^6^ However, despite these advances, there remains a significant gap in understanding the experiences and perspectives of physiotherapists working in this field^10^ Most literature focuses on treatment outcomes and patient-reported measures, while the professional challenges, educational needs, and emotional demands faced by practitioners are largely overlooked^11^^,^^12^ Without this insight, developing targeted training programs and improving clinical service delivery remain difficult^9^^,^^10^

Limited evidence exists on how physiotherapists navigate everyday clinical practice in this emerging field, including how they construct professional roles, address sociocultural sensitivities related to masculinity, and engage with multidisciplinary care processes. Qualitative exploration of physiotherapists’ practice-based experiences is therefore essential to understand not only the nature of existing challenges, but also how these challenges shape clinical decision-making and service delivery. This study aimed to explore physiotherapists’ experiences and perspectives on clinical practice in men’s health physiotherapy, and to examine how sociocultural norms and healthcare system structures shape their professional practice.

## Materials and methods

### Design

The study employed a qualitative design using reflexive thematic analysis to explore the lived experiences of the participants. Semi-structured, in-depth interviews were utilized as the primary data collection method. This approach allowed for flexibility to explore emerging topics in depth while ensuring key research questions were addressed. All interviews were conducted individually. During the interviews, the researcher not only asked pre-determined questions from an interview guide but also posed additional probing questions to elaborate on participants' responses without leading them or altering their intended meaning^13^ The design and reporting of this study were guided by the Standards for Reporting Qualitative Research (SRQR)^14^

### Participants

Purposive sampling was used to recruit male physiotherapists with specific experience in men's health physiotherapy. All participants included in the study were male physiotherapists currently practicing in the field of men's health physiotherapy. Methodological guidance suggests that qualitative studies may achieve sufficient depth with sample sizes typically ranging from 5 to 25 participants,^15^ and data saturation is considered a key indicator for determining sample size adequacy^16^ Potential participants across Türkiye were identified through purposive sampling via professional networks and publicly available sources and contacted via email and social media (WhatsApp, Instagram). Interviews with the first 15 physiotherapists who responded positively to the invitation were conducted sequentially and subsequently analyzed. Thematic saturation was reached by the 10th interview, with no new subthemes or substantive analytic insights emerging after the seventh interview (interviews 8‒10), and the thematic framework remaining conceptually stable. Recruitment was therefore discontinued^17^ Information about the participant physiotherapists is provided in Table 1.

**Table 1:** Information about physical therapists.

	Age	Education level	Professional experience (years)	Experience in men's health physiotherapy (years)	Total number of patients followed up in men's health physiotherapy
**P01**	32	Bachelor's degree	9	3	50
**P02**	42	Bachelor's degree	18	4	40
**P03**	23	Bachelor's degree	1	1	20
**P04**	44	Master's degree	21	4	300‒350
**P05**	32	Master's degree	10	5	100
**P06**	30	Bachelor's degree	8	3	30‒40
**P07**	31	Master's degree	9	4	40
**P08**	33	Bachelor's degree	9	5	75
**P09**	32	Doctorate degree	10	8	250
**P10**	39	Doctorate degree	15	10	400‒450

### Data collection

Between February and March 2025, a total of 10 semi-structured, in-depth interviews were conducted with 10 physiotherapists in Türkiye. Each interview lasted between 30- and 45-minutes and was conducted online via the Zoom application. Written informed consent was obtained from all participants, and the collected data were securely stored with identifiers removed to ensure confidentiality. The interview guide consisted of broad, open-ended prompts designed to explore participants’ perspectives and experiences related to men’s health physiotherapy (Appendix 1). In line with an exploratory qualitative approach, interviews were conducted flexibly, with participants encouraged to elaborate on issues they considered most relevant. Probing questions were used to explore meanings, experiences, and tensions rather than to confirm predefined assumptions. Early interviews prompted minor refinements to question wording and sequencing to enhance clarity, while the core open-ended structure was retained throughout data collection^18^

### Data analysis

The data were analyzed using Braun and Clarke's six-phase reflexive thematic analysis approach^19^ Analysis began with close engagement with the data through transcription and repeated reading, which allowed the researchers to become familiar with patterns of meaning across interviews. Initial codes were generated inductively through line-by-line coding, capturing participants' expressed experiences and underlying meanings. These codes were then examined for relationships and similarities, leading to the development of preliminary themes that reflected shared experiential patterns rather than isolated statements.

Throughout the analytic process, emerging themes were continuously reviewed against the coded data and the full data set, allowing refinement of theme boundaries and interpretive focus. Analytic attention was directed not only to convergences but also to tensions and contradictions within participants' accounts. Themes and sub-themes were finalized through iterative team discussions, where alternative interpretations were critically examined, and consensus was reached on thematic definitions and boundaries^20^^,^^21^ When divergent interpretations emerged, these were addressed through reflexive discussion and re-examination of the data, with attention to maintaining analytic coherence rather than achieving agreement based on frequency or majority opinion. Verbatim quotations were used to illustrate and substantiate the interpretive claims derived from the analysis.

The coding process was managed using MAXQDA software (version 2020.2.2). The researcher (B.Ö.) performed the initial line-by-line coding and developed a preliminary coding framework. This inductive process was complemented by discussions with the research team and comparative analysis of codes and themes across interviews^22^ Thematic saturation was confirmed by the final interviews, as no new analytic insights relevant to the study aims emerged, and the thematic framework remained conceptually stable. The sequential development of themes is presented in Supplementary Table S1^23^ In addition, the coding framework and code frequencies are presented in Supplementary Table S2, and the distribution of themes and subthemes across participants is illustrated in Supplementary Figure S1.

### Researcher reflexivity and positionality

This study was conducted by a team of three physiotherapist-academics in Türkiye. One author (U.E.) has clinical experience in men’s health physiotherapy, while the other two (B.Ö. and S.U.) do not practice in this subspecialty but contribute expertise in rehabilitation sciences and qualitative research, providing a balance between insider and external perspectives. Consistent with reflexive thematic analysis, researchers maintained ongoing reflexive awareness rather than attempting to bracket their positions. Primary coding was undertaken by B.Ö., with analytic decisions discussed across the team to critically examine interpretations. The combination of clinical insight and external questioning supported attention to tensions within the data, enhancing transparency and interpretive credibility.

### Ethical statement

The study was approved by the Bingöl University Ethics Committee for Scientific Research and Publications in Health Sciences (Decision n° 2025/06/02, dated 06.02.2025). In addition, all participants provided written informed consent before enrolment in the study. Furthermore, the study was conducted according to the Declaration of Helsinki of 1964, as revised in 2013.

## Results

Thematic analysis generated an overarching interpretive framework capturing participants’ descriptions of men’s health physiotherapy as a field shaped by the simultaneous presence of professional opportunity and structural constraint. Participants described men’s health physiotherapy as professionally meaningful and future-oriented, offering opportunities for clinical impact, professional differentiation, and moral engagement with unmet patient needs. At the same time, these experiences were embedded within broader sociocultural and systemic conditions, including masculinity-related stigma, limited interdisciplinary integration, and structural barriers within healthcare service organizations. Together, these accounts reflected a dynamic and contextually shaped professional landscape rather than a uniformly positive or negative practice experience.

This interpretive framework was articulated through five interrelated themes: 1) *Entering an Emerging Field: Professional Identity Formation*; 2) *Negotiating Masculinity, Stigma, and Clinical Trust*; 3) *Practicing Holism within Structural Constraints*; 4) *Systemic Isolation in a Multidisciplinary Care Ideal*; and 5) *Imagining Professional Legitimacy and Sustainability.* These themes should not be interpreted as discrete or independent categories, but as interconnected dimensions of participants’ experiences that collectively illustrate how men’s health physiotherapy is practiced, negotiated, and conceptualized within a developing professional and healthcare context.

Theme development followed an iterative and interpretive analytic process in which initial descriptive codes were progressively grouped and refined to capture shared experiential patterns rather than frequency of occurrence. Early coding generated a broad range of practice-related codes, including career motivations, patient interaction dynamics, stigma-related challenges, clinical practice adaptations, and system-level barriers. Through ongoing comparison and conceptual grouping, these codes were organized into higher-order themes representing underlying processes shaping physiotherapists’ professional experiences. For example, early codes related to curiosity, professional opportunity, and recognition of unmet patient needs were initially examined as separate descriptive elements. Further analysis revealed that these codes collectively reflected a broader process of negotiating professional identity within an emerging and under-institutionalized field, contributing to the development of the theme *Entering an Emerging Field: Professional Identity Formation*. Similarly, codes associated with stigma, privacy concerns, and patient hesitation were interpreted as interconnected sociocultural processes influencing therapeutic relationships, informing the theme *Negotiating Masculinity, Stigma, and Clinical Trust.*

Later stages of analysis focused on identifying tensions and contradictions within participants’ accounts. Participants simultaneously described men’s health physiotherapy as enabling a holistic clinical perspective while also reporting structural limitations in multidisciplinary collaboration. Rather than treating these accounts as inconsistent, they were interpreted as reflecting a systemic paradox within practice contexts, contributing to the development of the themes *Practicing Holism within Structural Constraints and Systemic Isolation in a Multidisciplinary Care Ideal*. Finally, participants’ forward-looking recommendations were interpreted as expressions of efforts to secure professional legitimacy and long-term sustainability within a developing field, informing the theme *Imagining Professional Legitimacy and Sustainability.*

***1. Theme: entering an emerging field: professional identity formation***

Physiotherapists’ entry into men’s health physiotherapy can be understood as an active process of professional identity formation rather than a purely pragmatic career choice. Participants’ accounts suggest that engagement with this emerging field involved navigating uncertainty while simultaneously seeking professional distinction and meaning. The convergence of personal curiosity, perceived gaps in patient care, and opportunities within an under-institutionalized specialty positioned entry into men’s health physiotherapy as both a professional risk and a form of future-oriented investment. In this sense, choosing to work in men’s health physiotherapy reflected not only individual motivation but also a strategic negotiation of professional identity within a field lacking established boundaries and formal recognition.

Sub-theme 1.1: Curiosity as Professional Risk-Taking: Participants’ expressions of curiosity can be interpreted as a form of professional risk-taking, whereby interest in an unfamiliar and weakly institutionalized field involves uncertainty alongside the potential for future professional differentiation. Rather than reflecting passive interest, curiosity functioned as an active orientation toward possibility in a context marked by limited training pathways and unclear career trajectories. Engaging with men’s health physiotherapy thus required participants to tolerate ambiguity while imagining long-term professional rewards associated with pioneering work in an emerging specialty. One participant articulated this process as follows: “*While researching, I came across men's health rehabilitation*… *I became curious. When I saw that the field and employment opportunities in this area were limited in our country, it piqued my interest even more. I decided to work in this field*.” (P09).
Sub-theme 1.2: Moral Responsibility Toward Unmet Needs: Participants’ accounts suggest that entry into men’s health physiotherapy was frequently shaped by a perceived moral responsibility to respond to unmet clinical needs. Awareness of the high prevalence of untreated pelvic health problems among men was not described merely as an epidemiological observation, but as an ethical call to action that conferred purpose and meaning on professional choices. This moral orientation positioned participants as advocates for a marginalized patient group, reinforcing their commitment to work in a field where care was perceived as both necessary and insufficiently provided. For some participants, this sense of responsibility was further intensified by personal or vicarious experiences of treatment effectiveness, which strengthened their belief in the value of intervention and legitimized their professional engagement in this area. One participant described this process as follows: “*I realized that the number of patients with pelvic floor problems was very high, and there was a need… Slowly, I began to focus on working in this field*.” (P02). For some, this awareness was personal, having experienced the benefits of treatment themselves.
Sub-theme 1.3: Strategic Positioning in an Under-Institutionalized Field: Participants interpreted the limited number of specialists not only as a service gap but as an opportunity for strategic professional positioning within an under-institutionalized field. Entering men’s health physiotherapy was framed as a deliberate choice to gain early expertise, professional visibility, and future security in a domain with weak competition and unclear boundaries. This positioning reflects an entrepreneurial orientation toward specialization, where uncertainty was balanced against the anticipated symbolic and career-related rewards of pioneering practice. “*Sometimes it's good to be a pioneer… Since the number of people working in this field is low, I think those working in this field have a clear future ahead*.” (P08). Another participant added, “*Instead of trying to fit into existing fields*… *I thought it would be easier and more rewarding to make a difference in a field that had not been worked on before.*” (P03).

***2. Theme: negotiating masculinity, stigma, and clinical trust***

A central dimension of physiotherapists’ experiences in men’s health physiotherapy involved the ongoing negotiation of masculinity-related norms, sociocultural stigma, and the establishment of clinical trust. Participants’ accounts indicate that patient engagement was rarely straightforward and was often shaped by deeply embedded cultural meanings attached to male bodies, intimacy, and vulnerability. Building trust, therefore, emerged not as a preliminary step but as a continuous relational process through which physiotherapists sought to legitimize both the therapeutic intervention and their own professional role within a sensitive clinical context.

Sub-theme 2.1: Sociocultural and Masculinity-Related Stigma: Participants described sociocultural stigma as a primary barrier shaping men’s engagement with pelvic health physiotherapy, particularly when interventions were perceived as challenging dominant norms of masculinity. Internal assessment and treatment procedures were frequently interpreted by patients as threats to bodily autonomy, masculinity, and moral integrity, positioning physiotherapy not merely as a clinical intervention but as a culturally charged encounter. Within this context, stigma functioned as a powerful gatekeeping mechanism that influenced patients’ willingness to seek care, disclose symptoms, and sustain treatment participation. “*Especially after men hear about internal procedures, they tend to have issues with their masculinity and pride. Therefore, they can often behave with prejudice towards the treatment. I've even heard words like rape and harassment*.” (P05).
Sub-theme 2.2: Hesitation and Privacy Concerns: Participants described patient hesitation and privacy concerns as behavioral manifestations of limited awareness regarding the role of physiotherapy in men’s health. Shyness and doubt were not interpreted as individual reluctance alone, but as responses shaped by uncertainty about bodily exposure, treatment legitimacy, and professional boundaries. In this sense, privacy concerns functioned as an initial protective strategy through which patients managed perceived vulnerability in an unfamiliar and culturally sensitive therapeutic context. “*Those who have no prior knowledge*… *initially experience astonishment and may be shy*.” (P05). Others approached with skepticism after failed previous treatments: “*Some patients may approach with doubt because they have been exposed to many treatment protocols before. They might not believe it could be beneficial*.” (P10).
Sub-theme 2.3: The Transition to Trust and the “Rescuer” Perception: Participants described a marked relational shift in the therapist-patient dynamic once initial stigma and hesitation were overcome. Through sustained communication and observable clinical improvement, trust gradually replaced doubt, enabling patients to reframe physiotherapy as a legitimate and effective form of care. Within this process, physiotherapists were sometimes positioned as “rescuers”, not solely because of personal competence, but because they represented a non-invasive alternative in a healthcare context where surgical or pharmacological options were often perceived as the only available solutions. This reconfiguration of trust was experienced as professionally affirming, reinforcing participants’ sense of purpose and legitimacy within a sensitive clinical domain. “*As they see faster results, they begin to view men's health physiotherapy and physiotherapists as rescuers*.” (P09). This transition was described as a highly rewarding aspect of their work.

***3. Theme: practicing holism within structural constraints***

Participants described several aspects of men’s health physiotherapy as professionally rewarding; however, these perceived advantages were consistently shaped by broader structural constraints. Job satisfaction and a sense of professional uniqueness did not stem solely from clinical outcomes, but from the ability to enact a holistic approach in contexts where systemic support and interdisciplinary integration were limited. Holism thus emerged not as an inherent feature of the healthcare system, but as an individually sustained practice that carried both professional meaning and implicit strain.

Sub-theme 3.1: Individually Practiced Holism: Participants framed holism as a central professional gain, describing an expanded clinical perspective that integrated pelvic health with broader musculoskeletal assessment and management. However, this holistic approach was enacted primarily at the individual level rather than being supported by structured interdisciplinary systems. Holism, therefore, functioned as a personally sustained clinical practice, reflecting both professional growth and a compensatory response to limited systemic integration. “*To be honest, I can say that my perspective on patients and cases*… *has expanded to a much broader view. It has helped me adopt a more holistic approach*.” (P06).
Sub-theme 3.2: Reduced Physical Burden and Career Longevity: Participants perceived men’s health physiotherapy as less physically demanding compared to other physiotherapy specialties, a characteristic that was closely linked to considerations of long-term career sustainability. Reduced physical strain was not framed merely as comfort, but as a pragmatic advantage that enabled participants to envision prolonged clinical engagement without the risk of occupational exhaustion or injury. In this sense, physical manageability functioned as a structural factor shaping professional longevity within an otherwise demanding healthcare environment. “*And it's physically less exhausting. Compared to many other fields, it's much less tiring*.” (P04). This was seen as beneficial for long-term career sustainability.
Sub-theme 3.3: Professional Visibility and Symbolic Capital: Participants described specialization in men’s health physiotherapy as a source of professional visibility and symbolic capital within a competitive healthcare market. Working in a novel and weakly populated field enhanced distinctiveness, allowing physiotherapists to differentiate themselves from peers and gain recognition, particularly in private practice settings. This form of visibility extended beyond economic advantage, contributing to perceived professional legitimacy and reinforcing participants’ sense of value within an emerging specialty. “*Being a specific field is, in my opinion, a great advantage*… *It also creates an advantage in terms of working in the private sector and competition*.” (P09).

***4. Theme: systemic isolation in a multidisciplinary care ideal***

Beyond challenges arising from patient engagement, participants described systemic conditions that fundamentally shaped and constrained their clinical practice. Although men’s health physiotherapy was consistently conceptualized as inherently multidisciplinary, participants’ accounts revealed a structural reality characterized by professional isolation, fragmented referral pathways, and limited interprofessional collaboration. This disjuncture between the ideal of integrated care and the realities of practice positioned physiotherapists as individually responsible for managing complex clinical needs that exceeded the formal boundaries of their professional role.

Sub-theme 4.1: Educational and Awareness Barriers Among Patients: Participants identified limited patient awareness as a critical structural barrier that complicated rehabilitation processes, particularly in relation to pelvic floor muscles, which were often described as “invisible” and difficult to conceptualize. Difficulties in understanding where and how to activate these muscles were interpreted not as individual failure, but as the outcome of absent public education and minimal prior exposure to pelvic health concepts. This knowledge gap frequently delayed effective engagement with rehabilitation and contributed to prolonged symptom duration. Moreover, participants described a pattern of late presentation, whereby patients sought care only after symptoms had become persistent or severe, further increasing treatment complexity. In this sense, limited awareness functioned as part of a broader systemic cycle that intensified clinical demands and reinforced physiotherapists’ individual responsibility within an under-supported care structure. “*Unfortunately, because patients have never focused on this area before, they don't know exactly where to contract and how to do the exercise at first*.” (P10). “*There are those who continue living with these complaints… This leads to the condition worsening*.” (P07).
Sub-theme 4.2: Structural Barriers to Collaborative Care: Participants consistently identified the absence of structured multidisciplinary collaboration as a major constraint on clinical effectiveness. Despite conceptualizing men’s health physiotherapy as inherently multidisciplinary, participants described a siloed healthcare environment in which formal collaboration with urologists, psychologists, and other specialists was limited or informal. This structural fragmentation restricted coordinated care pathways and placed the burden of integration disproportionately on individual physiotherapists. The lack of collaboration was thus interpreted not as a failure of professional intent, but as a systemic limitation embedded within the organization of care. As a result, the delivery of comprehensive, biopsychosocial treatment remained aspirational rather than operational, reinforcing experiences of professional isolation within an otherwise multidisciplinary care ideal. “*I think this is a very multidisciplinary approach… Unfortunately, I think it is a somewhat lacking situation, especially in our country*.” (P07).

***5. Theme: imagining professional legitimacy and sustainability***

This theme captures how participants’ recommendations reflect an effort to imagine and secure professional legitimacy and long-term sustainability within an emerging and structurally constrained field.

Sub-theme 5.1: Therapeutic Communication as Boundary Work: Advanced communication skills were framed not merely as a desirable competency, but as a form of boundary work through which physiotherapists negotiate stigma, establish trust, and legitimize their role within sensitive clinical encounters. “*Especially for physiotherapists considering working in this field, effective interpersonal skills and communication are crucial*.” (P08).
Sub-theme 5.2: Technology as a Compensatory Strategy: The use of biofeedback and sensory technologies was described as a compensatory response to both patients’ limited bodily awareness and the absence of structured educational pathways. Technology thus functioned as a mediating tool that translated abstract bodily processes into tangible clinical experiences. “*Therefore, I frequently use sensory input and biofeedback to help patients understand the exercises*.” (P01).
Sub-theme 5.3: Imagining Multidisciplinary Care Beyond Structural Absence: Calls for multidisciplinary collaboration reflected less an existing reality than an aspirational response to professional isolation. Participants articulated a vision of integrated care as a means of redistributing clinical responsibility and addressing dimensions of men’s health that exceeded the scope of physiotherapy alone. *“In cases of erectile dysfunction, I believe multidisciplinary work is absolutely crucial*… *There may be a psychological foundation, and they definitely need to address this with a sexual therapist*.” (P06).

## Discussion

Men’s health physiotherapy in Türkiye is emerging as a professionally meaningful and promising area of specialization, while simultaneously being shaped by strong sociocultural norms and structural constraints. The findings indicate that men’s health physiotherapists construct their professional identities at the intersection of opportunities such as pioneering roles, clinical satisfaction, and professional visibility, and barriers, including masculinity norms, stigma, and limited multidisciplinary collaboration. Men’s health physiotherapy therefore, represents a field in which the ideal of holistic care is largely sustained through individual clinical effort rather than institutionally embedded within structured systems of interdisciplinary support. This study thus conceptualizes men’s health physiotherapy not merely as a developing clinical subspecialty, but as a professional identity domain formed within the organizational dynamics of the Turkish healthcare system and prevailing cultural norms, where opportunity and constraint coexist.

### Navigating a new professional frontier

The findings suggest that entry into men’s health physiotherapy should be understood not as a simple career choice, but as an active process of professional identity construction within a field that remains only partially institutionalized. Participants’ career decisions were shaped at the intersection of perceived clinical gaps and strategic professional positioning. As Dent et al. argue in their discussion of professional field formation in healthcare, new areas of specialization often emerge in response to unmet demand and the opportunities such demand creates for differentiation^24^ In this sense, the limited number of specialists in men’s health physiotherapy was experienced not merely as a service deficit, but as a structural opening through which physiotherapists could develop niche expertise and symbolic professional distinction.

Participants’ emphasis on curiosity and “being pioneers” (P08) reflects more than individual interest; it signals a deliberate willingness to assume uncertainty in pursuit of professional visibility within a specialty whose boundaries are still evolving. At the same time, this positioning cannot be explained solely in entrepreneurial terms. Participants repeatedly highlighted unmet patient needs, particularly in areas such as pelvic health and post-prostatectomy rehabilitation, which are known to significantly affect quality of life^1^^,^^25^ Specialization in this field, therefore, appears to be grounded not only in strategic calculation but also in a sense of ethical responsibility toward underserved patient populations.

However, the very structural features that make the field attractive, namely limited institutional recognition, weak interdisciplinary integration, and relatively low competition, also indicate that men’s health physiotherapy has not yet achieved full institutional embedding within the healthcare system. Entry into this field thus involves negotiating opportunity alongside structural ambiguity. The organization of the Turkish healthcare system further shapes how this dual positioning is experienced in practice. In settings where referral mechanisms and subspecialty structures remain only partially formalized, professional identity formation in men’s health physiotherapy is influenced not only by increasing clinical demand but also by systemic gaps and organizational constraints. Early engagement in this field, therefore, reflects not only individual career development, but also participation in the ongoing construction of a specialty whose professional legitimacy and boundaries remain under development.

### Confronting the weight of masculinity

One of the most structurally significant findings of this study is that sociocultural norms shaped around masculinity influence not only patient behavior but also the professional positioning of men’s health physiotherapists. The literature on men’s health-seeking behaviors identifies restrictive emotionality, fear of stigma, and the association of help-seeking with weakness as primary barriers^26^, ^27^, ^28^ The “issues related to masculinity” described by participants (P05) should therefore be understood not as individual preferences, but as reflections of deeply embedded cultural scripts that equate vulnerability with weakness^29^^,^^30^ According to participants’ accounts, rectal pelvic floor assessment and intervention procedures in particular conflict with norms related to male bodily autonomy and privacy, contributing to delays in seeking treatment.

The fact that all participants in this study were male physiotherapists adds an additional interpretive layer to these findings. In a context where patriarchal norms strongly structure male identity, participants occupy a dual position: they are both men situated within this cultural framework and healthcare professionals attempting to normalize the expression of vulnerability among male patients. These findings indicate that the therapeutic relationship is not merely a clinical interaction but also a culturally mediated process. In situations where masculinity norms restrict help-seeking behaviors, physiotherapists assume an active role in rebuilding trust and legitimizing intervention.

In the Turkish healthcare and sociocultural context, sexuality and pelvic health remain highly sensitive topics, often surrounded by privacy concerns and social taboos, which further intensifies this tension in clinical practice. Traditional masculine ideals such as control, self-sufficiency, and emotional restraint can increase resistance to adopting the sick role. When combined with economic, cultural, and communication barriers, these norms contribute to delayed help-seeking behavior. Existing literature suggests that autonomy-supportive approaches, critical engagement with harmful masculine norms, and strengthened patient-provider communication may help reduce such delays^31^^,^^32^ However, the present findings suggest that men’s health physiotherapists are not merely implementers of such strategies; they are actors who continuously negotiate cultural norms within clinical encounters. Their role, therefore, extends beyond technical intervention to encompass a form of cultural mediation. Participants’ efforts to “normalize men expressing their vulnerabilities” require actively engaging with and, at times, challenging the manifestations of patriarchal norms within healthcare settings. Consequently, stigma and masculinity norms function not only as constraints on patient behavior but also as significant contextual forces shaping how physiotherapists define their professional roles within this emerging field.

### The holistic advantage and the collaboration deficit

Participants emphasized the holistic approach as one of the primary advantages of men’s health physiotherapy. The ability to integrate pelvic health with musculoskeletal assessment aligns with contemporary rehabilitation principles in the management of complex and multifactorial conditions^33^^,^^34^ However, the findings suggest that this holism is not institutionally embedded within system-level structures, but rather sustained through individual clinical effort.

Participants also reported limited multidisciplinary collaboration, noting the absence of regular and structured integration with urologists and psychologists. Yet conditions such as erectile dysfunction and chronic pelvic pain, which have strong biopsychosocial components, inherently require coordinated interdisciplinary care to achieve truly holistic management^3^^,^^9^ In this sense, the situation observed in this study may be conceptualized not as institutionalized holism, but as a form of “isolated holism”. Isolated holism refers to a configuration in which clinicians adopt a broad, integrative perspective, yet operate without systematic support from structured interdisciplinary networks. This dynamic generates a tension between professional satisfaction and structural vulnerability. Physiotherapists position themselves as comprehensive practitioners, while simultaneously assuming individual responsibility for managing complex cases in the absence of formalized collaboration.

In Türkiye, this tension is closely related to the organization of the healthcare system. Limited referral mechanisms, the predominance of private-sector service delivery, and insufficiently structured channels for interdisciplinary communication constrain the institutional development of holistic care. Similar collaboration deficits have been identified as systemic challenges in other areas of rehabilitation^11^^,^^12^^,^^35^ However, as demonstrated in this study, within men’s health physiotherapy, such constraints affect not only service delivery but also the way professional identity is experienced and enacted. What is therefore at stake is not merely a lack of collaboration, but the structural limitation of the holistic care ideal itself. Holism is upheld as a professional value, yet in practice, it is sustained primarily at the individual level due to insufficient systemic support. This condition simultaneously reveals both the developmental potential and the structural fragility of men’s health physiotherapy as an emerging field.

### Contextualizing practice within the Turkish healthcare system

The healthcare system in Türkiye is formally structured as a tiered model based on a referral chain. However, in practice, mandatory gatekeeping mechanisms remain limited, and patients are often able to access secondary and tertiary care services directly^36^^,^^37^ This structural configuration operates within a mixed public-private system in which the private sector has gained increasing prominence over time. The growing weight of private-sector provision and a patient-driven access model may complicate the systematic institutionalization of interdisciplinary coordination at the organizational level.

Furthermore, in public healthcare institutions, access to physiotherapy services is typically contingent upon referral by a specialist physician in physical medicine and rehabilitation. The inability of physiotherapists to accept patients independently can result in interdisciplinary collaboration being shaped largely by informal, clinician-level relationships rather than by structured institutional pathways. Such regulatory arrangements may constrain professional autonomy, particularly in emerging subspecialties such as men’s health physiotherapy that have not yet achieved full institutional consolidation. Consequently, the systematic construction of multidisciplinary care becomes more difficult to operationalize.

Against this backdrop, the collaboration deficits described by participants cannot be attributed solely to professional reluctance; they are also embedded in structural features of the healthcare system. The tension observed between the “multidisciplinary ideal” and practical experiences of professional isolation in men’s health physiotherapy cannot be fully understood without reference to Türkiye’s healthcare delivery model and the formal boundaries of professional authority.

### Limitations

This study has three key limitations. First, the sample consisted exclusively of male physiotherapists. While this homogeneity enabled an in-depth exploration of male clinicians' perspectives, it may limit transferability to female physiotherapists practicing in the same field, as therapist gender may shape experiences of stigma, communication, and professional identity in ways not captured here. Second, the study was conducted within the sociocultural and organizational context of the Turkish healthcare system. Cultural norms surrounding masculinity and structural characteristics of healthcare delivery in Türkiye may have influenced participants' experiences. Therefore, the findings should be interpreted within this contextual framework and may not be directly transferable to other settings. Third, the interview guide was not formally pilot tested. Although early interviews allowed for minor iterative refinements to question wording and sequencing, the absence of a dedicated pilot phase may have constrained the optimization of certain prompts. Systematic pilot testing is recommended for future studies.

## Conclusion

This study reveals that men's health physiotherapy in Türkiye has been shaped by a persistent tension between professional opportunities and structural constraints. Participant narratives show that the orientation towards this emerging field is not limited to responding to unmet clinical needs, but also involves the active negotiation of professional identity in a field where institutional recognition and interdisciplinary integration are not yet fully established.

One of the main contributions of this study is that it makes visible a form of holism pursued at the individual level. While participants emphasise the value of a holistic approach, the lack of structured multidisciplinary collaboration and formalised referral mechanisms limits the systematic implementation of integrated care. Consequently, holistic practice is mostly sustained at the level of individual clinicians rather than being supported by an institutional infrastructure. This situation shapes professional experience within a dual structure that encompasses both autonomy and isolation.

Furthermore, these findings should be evaluated within the sociocultural and organisational context of the Turkish healthcare system. Masculinity norms, referral structures, and institutional arrangements directly influence both help-seeking patterns and professional practice. In this context, physiotherapists working in men’s health are not merely professionals providing clinical services; they are also positioned as relationship brokers who negotiate stigma, build legitimacy, and establish trust in culturally sensitive interactions.

Overall, the findings provide a contextually grounded understanding of how professional identity and structural conditions shape the development of men’s health physiotherapy. Strengthening interdisciplinary collaboration, expanding educational pathways, and formalising referral processes may support the transition from individually sustained holistic practice toward more integrated and institutionally supported models of care.

## Authors' contributions

Umit Erkut: Conceptualization; data acquisition; study design; clinical reviewing; final approval.

Birol Önal: Conceptualization; study design; writing, editing and clinical reviewing the manuscript; final approval.

Sefa Unes: Conceptualization; writing, editing and clinical reviewing the manuscript; project administration; final approval.

## Funding

The authors received no financial support for the research, authorship, and/or publication of this article.

## Declaration of competing interest

The authors declared no potential conflicts of interest with respect to the research, authorship, and/or publication of this article.

## Figures and Tables

**Table:** 

1. Why did you decide to work in men's health?
2. What are the advantages of working in men's health?
3. What are the disadvantages of working in men's health?
4. How do patients view men's health physiotherapy?
5. What are your experiences during the session? What is the most interesting event you have experienced?
6. What are your recommendations for physiotherapy in men's health?
7. What do you think are the situations where physiotherapy is useful in men's health? What are the situations where you think it is not useful?
8. What is the importance of working with a multidisciplinary team in the rehabilitation process in men's health? What are your experiences with this kind of collaboration process?

## Data Availability

The data that support the findings of this study are available from the corresponding author, [UE], upon reasonable request.

## Appendix B Supplementary materials

Image, application 1
