# Supplementary material for: The scope of physiotherapy in men’s health: a qualitative study on clinician experiences
Source: Clinics (Sao Paulo). 2026 Jul 1;81:101039. doi: 10.1016/j.clinsp.2026.101039 (PMC13334809; doi:10.1016/j.clinsp.2026.101039)

**CLINICS-D-25-002069_ Supplementary Material**

**Supplementary Table S1** Development of themes and saturation.

| **Interview** | **Participant** | **New theme(s) identified?** | **Number of new themes** | **Cumulative themes** |
| --- | --- | --- | --- | --- |
| 1 | P01 | Yes | 6 | 6 |
| 2 | P02 | Yes | 3 | 9 |
| 3 | P03 | Yes | 2 | 11 |
| 4 | P04 | Yes | 1 | 12 |
| 5 | P05 | Yes | 1 | 13 |
| 6 | P06 | Yes | 1 | 14 |
| 7 | P07 | Yes | 1 | 15 |
| 8 | P08 | No | 0 | 15 |
| 9 | P09 | No | 0 | 15 |
| 10 | P10 | No | 0 | 15 |

**Note:** A total of 15 sub-themes were identified across 10-interviews. The first six interviews introduced 14-themes, with the final theme emerging in the 7^th^ interview. Interviews 8‒10 yielded no new themes, indicating that thematic saturation was achieved and confirmed.

**Supplementary Table S2** Coding framework.

| **MAXQDA Coding Framework** | **Frequency** |
| --- | --- |
| **Imagining Professional Legitimacy and Sustainability** |  |
| Therapeutic Communication as Boundary Work | **6** |
| Imagining Multidisciplinary Care Beyond Structural Absence | **10** |
| Technology as a Compensatory Strategy | **9** |
| **Negotiating Masculinity, Stigma, and Clinical Trust** |  |
| The Transition to Trust and the "Rescuer" Perception | **5** |
| Hesitation and Privacy Concerns | **11** |
| Sociocultural and Masculinity-Related Stigma | **9** |
| **Practicing Holism within Structural Constraints** |  |
| Reduced Physical Burden and Career Longevity | **3** |
| Professional Visibility and Symbolic Capital | **6** |
| Individually Practiced Holism | **9** |
| **Entering an Emerging Field: Professional Identity Formation** |  |
| Moral Responsibility Toward Unmet Needs | **4** |
| Curiosity as Professional Risk-Taking | **4** |
| Strategic Positioning in an Under-Institutionalized Field | **11** |
| **Systemic Isolation in a Multidisciplinary Care Ideal** |  |
| Educational and Awareness Barriers Among Patients | **27** |
| Structural Barriers to Collaborative Care | **13** |

**Supplementary Figure 1** Distribution of themes and subthemes across participants.


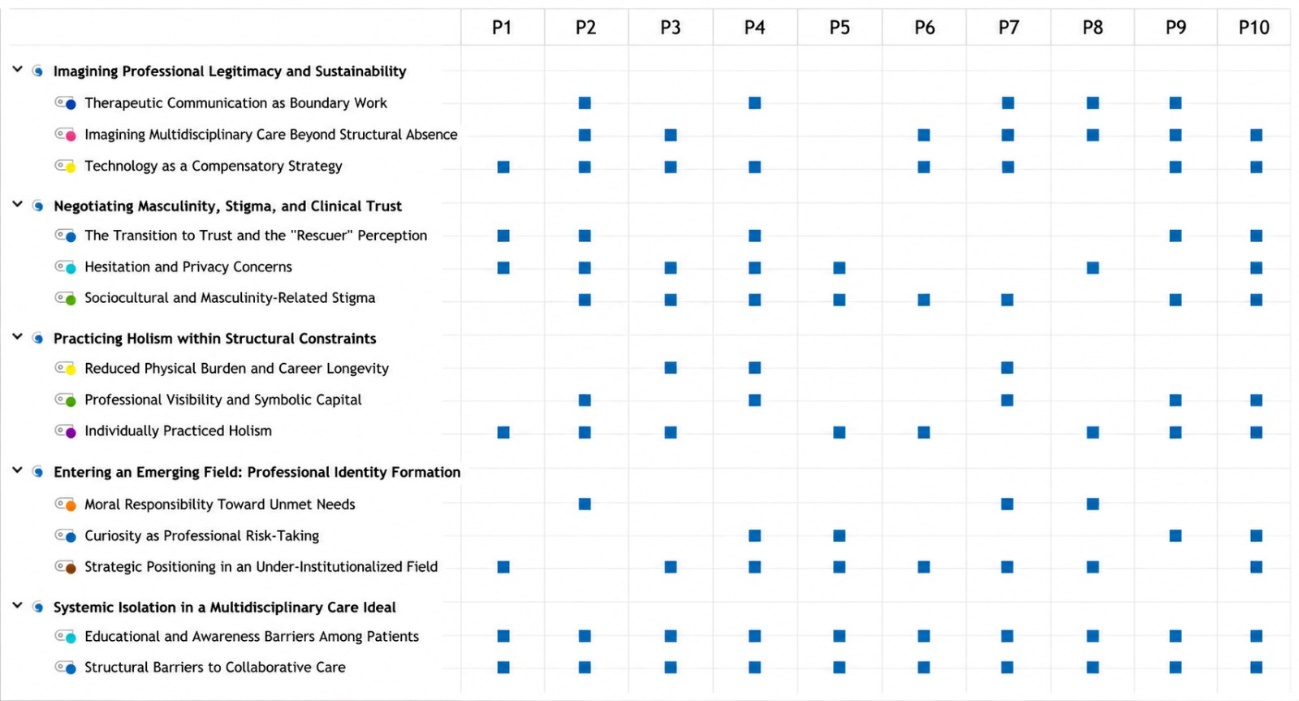

Supplement: Supplementary file 1 [file mmc1.docx]
